# Pathobiology of Obstructive Sleep Apnea-Related Dyslipidemia: Focus on the Liver

**DOI:** 10.1155/2013/687069

**Published:** 2013-01-02

**Authors:** Aibek E. Mirrakhimov, Alaa M. Ali

**Affiliations:** Department of Internal Medicine, Saint Joseph Hospital, 2900 North Lake Shore, Chicago, IL 60657, USA

## Abstract

Obstructive sleep apnea and dyslipidemia are common medical disorders that independently increase vascular morbidity and mortality. Current animal and human data show that, indeed, obstructive sleep apnea may mediate pathological alterations in cholesterol and triglyceride metabolism. The mechanisms involved are increased lipolysis, decreased lipoprotein clearance, and enhanced lipid output from the liver. Human evidence shows that the treatment of obstructive sleep apnea with continuous positive airway pressure leads to an improvement of postprandial hyperlipidemia. However, more studies are needed, to clarify the pathophysiology of the interrelationship between obstructive sleep apnea and dyslipidemia and whether treatment of obstructive sleep apnea will lead to an improvement in the lipid profile and, more importantly, reduce hyperlipidemia-related vascular outcomes.

## 1. Introduction

Obstructive sleep apnea (OSA) is a common medical disorder affecting up to 24% of the general US population [1]. The disorder is characterized by repetitive complete and/or partial collapse of the upper airways. OSA is strongly associated with vascular [2, 3], metabolic [4], and kidney diseases [5]. Therefore, OSA has to be approached not just as a simple snoring problem, but rather should be considered as a medical disorder with systemic features. This notion is supported by the fact that OSA treatment may improve the function of target organs [6].

Current evidence suggests that OSA disturbs fundamental biochemical processes and is associated with low-grade systemic inflammation and oxidative stress [7]. Indeed, this may underlie the fact of why individuals affected with OSA are at increased risk for comorbid diseases, particularly for vascular diseases. 

Dyslipidemia, on the other hand, is the group of disorders of cholesterol (Ch) and/or triglyceride (TG) metabolism with a well-known detrimental impact on increased cardiovascular risk [8]. Furthermore, clinical evidence shows that OSA may be independently associated with dyslipidemia [9–18] and functional abnormalities of high-density lipoproteins (HDL) [19]. Moreover, OSA-targeted therapeutic intervention leads toward an improvement in the lipid profile [20–24]. However, others have failed to find any association between OSA and dyslipidemia in humans [25]. Differences in research methodology and the studied population may explain these conflicting results in clinical research on OSA and dyslipidemia.

The goal of this paper is to summarize the current knowledge on the pathogenesis of the potential interrelationship between OSA and dyslipidemia. Firstly, we will briefly overview the metabolism of Ch and TG. Secondly, we will discuss the data on increased lipid delivery to the liver in OSA models, including data on increased lipolysis. Thirdly, data on abnormal lipid clearance in OSA will be reviewed. Finally, we will discuss the evidence regarding how OSA may increase lipid synthesis in the liver.

## 2. Overview of Cholesterol and Triglyceride Metabolism

A detailed discussion of Ch and TG metabolism is beyond the scope of this paper and can be found elsewhere [26]. The aim of this section is to help the reader better understand the biochemistry of Ch and TG metabolism and to apply it to the pathogenesis of OSA-related dyslipidemias.

There are two main pathways of lipid metabolism: exogenous and endogenous. We will briefly review the exogenous pathway first, and then discuss the endogenous one.

The endogenous lipid pathway starts from the intestinal absorption of dietary TG and Ch, which will be bound to locally synthesized (small intestine) chylomicrons. Chylomicrons contain apolipoprotein (apo) B48 and will acquire apo C II and apo E in the bloodstream from other lipoprotein particles, particularly from HDL. Apo C II serves as a ligand for the enzyme lipoprotein lipase (LPL), which is located predominantly in the adipose tissue. LPL will hydrolase the TG content of chylomicrons to form glycerol and free fatty acids (FFA), which will be taken up by adipocyte for storage. Subsequently, smaller chylomicron particles can transfer some proteins to HDL and finally be taken up by the liver for Ch and TG turnover.

The exogenous lipid pathway starts in the liver and is believed to be more clinically relevant to the initiation and progression of the atherosclerotic process. Similar to the endogenous pathway, the process starts with the formation of lipoproteins rich in TG, particularly, very low-density lipoproteins (VLDL). VLDL are smaller particles than chylomicrons and contain apo B100 instead of apo B48. In addition to apo B100, VLDL contain apo CII, apo C III, and apo E. Similarly, apo C II activates LPL for the hydrolyzation of the TG content, resulting in the formation of intermediate density lipoproteins (IDLs). IDLs can be either taken up by the liver through apo B 100 and apo E ligands or can be converted into low-density lipoproteins (LDLs) by hepatic lipase and cholesterol transfer from HDL. Thereafter, Ch can be used in bile acid synthesis, the production of steroid hormones, or can be taken up by macrophages via scavenger receptors with the subsequent formation of foam cells in the arterial bed. In addition to this LDL can be oxidized in the arterial wall and initiate proinflammatory cascade, which is known to be one of the major steps in the pathogenesis for atherosclerosis [27].

HDL, which is formed in the liver and intestine, contains apo A I, apo A II, apo A IV, apo C I–III, apo D, and apo E. Some of these apolipoproteins will be transferred to other particles, such as apo C II and apo E to chylomicrons. HDL is capable of removing Ch from tissues via apo A I and to a lesser extent via apo A-IV- and apo CI-mediated activation of the lecithin cholesterol acyl transferase (LCAT) enzyme. Ch carried on HDL can be transferred to apo B-containing lipoproteins. It is generally believed that HDL is protective against atherosclerosis and vascular disease; however, some controversy exists regarding the so-called protective role of HDL, mainly because no benefit is seen in terms of cardiovascular protection with increased HDL in clinical trials [28].

## 3. Pathogenesis of OSA-Related Dyslipidemia: Role of the Liver

### 3.1. Increased Lipid Delivery from the Adipose Tissue to the Liver

Repetitive episodes of apneas and hypopneas lead to a state of intermittent hypoxia (IH), which further alters a myriad of key biochemical processes [29]. Indeed, IH is able to increase gene expression of the hypoxia inducible factor (HIF) family with subsequent propagation of inflammation and an oxidative state [7]. Furthermore, IH increases sympathetic output [30], which may particularly explain why patients with OSA have a higher prevalence of arterial hypertension and vascular diseases [2]. 

Indeed, catecholamine hormones via their insulin-antagonizing effects modulate the activity of hormone sensitive lipase (HSL) in the adipose tissue, leading to the breakdown of TG into FFA and glycerol, which then will be resynthesized in the liver and form VLDL [31]. Barceló et al. showed that OSA patients have elevated levels of FFA, which can lead to increased cardiovascular and metabolic risk [32]. Moreover, an increased amount of nocturnal FFA was shown to be associated with accelerated cardiac disease progression in patient with OSA and concomitant heart failure [33].

Subsequent FFA influx into the liver may lead to the assembly of TG-rich VLDL and their further efflux into the systemic circulation [34]. Afterwards, VLDL will be converted into IDL by LPL, which can either be taken up by the liver for further lipid turnover or can accept cholesterol from HDL and become low-density lipoprotein (LDL) [35]. 

LDL cholesterol is notoriously known for its strong association with excessive cardiovascular morbidity and mortality [36]. Indeed, from a theoretical standpoint it is plausible that OSA- and IH-mediated upregulation of sympathetic nervous activity may decrease LDL clearance by blocking the activity of lipoprotein lipase.

### 3.2. Abnormal Lipid Clearance in OSA Models

As it was previously mentioned, lipoproteins rich in TG are cleared from the bloodstream via LPL-mediated hydrolysis. It is known that LPL activity is maintained by insulin and decreased by hormones such as cortisol and epinephrine that oppose the actions of insulin [37].

Jun et al. recently showed that IH downregulates TG clearance via a decrease in the activity of peroxisome proliferator-activated receptor-alpha (PPAR-*α*) [38]. PPAR-*α* is a nuclear receptor implicated in the regulation of transcriptional activity of multiple genes responsible for lipid metabolism, including LPL. CD 36 is another PPAR-*α*-regulated factor responsible for FFA uptake into cells. It has been shown that IH decreases CD36 and LPL activity via the downregulation of PPAR-*α* transcription [38].

A human study performed by Luyster et al. showed that OSA is associated with the LDL subclass B, which is known to be more resistant to clearance from the bloodstream [39]. Therefore, LDL subclass B has much more time to be oxidized and lead to vascular damage [39]. The authors speculated that insulin resistance associated with OSA leads to decreased activity of LPL and subsequent formation of LDL subclass B. On the top of that, as was mentioned previously, OSA may lead to increase in LDL and decrease in HDL via the stimulation of *α* adrenergic receptors [24].

Current scientific evidence suggests that OSA is associated with oxidative stress [7], which may lead to the oxidative modification of HDL, and subsequent dysfunction [19].

### 3.3. Increased Lipid Synthesis in OSA Models

On the molecular level, IH activates HIF, leading to a variety of fundamental alterations relevant to both physiological adaptation and disease [29]. Li et al. showed that IH increases the transcription of sterol regulatory element binding protein 1 (SREBP-1) and further activates stearoyl-coenzyme A desaturase 1 (SCD-1) in a mouse model of OSA [40]. SREBP-1 and SCD-1 are key factors responsible for TG assembly. The same research group confirmed the previous findings regarding the IH-mediated activation of SREBP-1 and SCD-1 [41]. However, no association was found between changes in SREBP-2 and hydroxy-3-methylglutaryl-CoA reductase (key factors in cholesterol biosynthesis) and IH.

Furthermore, researchers from Johns Hopkins University in the USA showed that the severity of IH is directly related to hyperlipidemia and liver oxidative stress in a mouse model of OSA [42]. Diet-induced dyslipidemia together with IH may lead to an upregulation in lipid synthesis as was shown in a C57BL/6J mouse model (these mice are relatively resistant to atherosclerosis) [43]. Supporting the role of SCD-1 in the development of dyslipidemia and atherosclerosis (at least in an animal model of OSA) is the fact that mice lacking functional SCD-1 have less dyslipidemia and atherosclerosis [44, 45].

## 4. Conclusion

**Figure 1:**
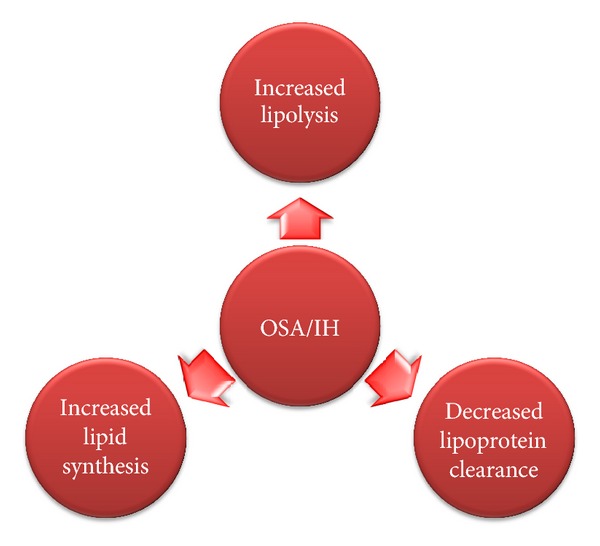
A simplified overview of OSA-mediated hyperlipidemia.

OSA and dyslipidemia are common medical disorders that independently increase vascular morbidity and mortality. Current animal and human data show that, indeed, OSA may mediate pathological alterations in cholesterol and triglyceride metabolism. The mechanisms involved are increased lipolysis, decreased lipoprotein clearance, and enhanced lipid output from the liver. A simplified overview of OSA-mediated hyperlipidemia is presented in Figure 1.

Human evidence shows that the treatment of OSA with continuous positive airway pressure leads to an improvement of postprandial hyperlipidemia. However, more studies are needed, to clarify the pathophysiology of the interrelationship between OSA and dyslipidemia and whether treatment of OSA will lead to an improvement in the lipid profile and, more importantly, reduce hyperlipidemia-related vascular outcomes.
